# Rich structure in the correlation matrix spectra in non-equilibrium steady states

**DOI:** 10.1038/srep40506

**Published:** 2017-01-17

**Authors:** Soham Biswas, Francois Leyvraz, Paulino Monroy Castillero, Thomas H. Seligman

**Affiliations:** 1 Instituto de Ciencias Físicas, Universidad Nacional Autónoma de México, Cuernavaca, México; 2 Centro Internacional de Ciencias, Cuernavaca, Mexico

## Abstract

It has been shown that, if a model displays long-range (power-law) spatial correlations, its equal-time correlation matrix will also have a power law tail in the distribution of its high-lying eigenvalues. The purpose of this paper is to show that the converse is generally incorrect: a power-law tail in the high-lying eigenvalues of the correlation matrix may exist even in the absence of equal-time power law correlations in the initial model. We may therefore view the study of the eigenvalue distribution of the correlation matrix as a more powerful tool than the study of spatial Correlations, one which may in fact uncover structure, that would otherwise not be apparent. Specifically, we show that in the Totally Asymmetric Simple Exclusion Process, whereas there are no clearly visible correlations in the steady state, the eigenvalues of its correlation matrix exhibit a rich structure which we describe in detail.

The analysis of correlation matrices has attracted considerable attention almost for a hundred years starting with multivariate analysis in finance^1^. In two pioneering papers Laloux *et al*. and Plerou *et al*. analysed a complex time signal—a time series of stock prices—and successfully disentangled the part due to chance and the systematic part via an analysis of the eigenvalues of the correlation matrix^2^,^3^,^4^. The same tools of correlation matrix analysis have recently gained attention from physicists in the discussion of critical phenomena and phase transitions: If we consider an extended system undergoing some kind of dynamics, the equal time correlations between the various components of the system yield a correlation matrix, the eigenvalues of which can be analysed. In this context, it has recently been shown^5^, that a power-law decay of correlations in space leads to a power-law behaviour for the large eigenvalues of the correlation matrix. We are thus led to ask whether the opposite is true. That is, does the observation of such power-law behaviour in the eigenvalues imply a power-law in spatial correlations? In a trivial sense, it is possible to find systems for which no correlations are apparent, and yet the power-law behaviour of the eigenvalues remains: We simply take a system which does display spatial power-law correlations, and “scramble” all of its components by subjecting them to an arbitrary permutation. Since this is a similarity transformation on the correlation matrix, this operation leaves the eigenvalues invariant, so that their power-law behaviour testifies to the existence of the spatial correlations, even though the latter have been masked by the random permutation. As an example, it is well-known that the correlations between the returns on different stocks are made far more apparent when these are ordered according to so called industry sectors. This can be seen, for example, in ref. 6.

However, we may ask whether there exist less trivial counterexamples. In the following, we shall suggest that there probably are: we shall analyse the so-called Totally Asymmetric Simple Exclusion Process, (TASEP) which shows little or no apparent spatial correlation, which additionally surely does not have a power-law decay. Yet in a given part of its phase diagram, which we shall describe shortly, this model displays a marked power-law feature in the spectra of its correlation matrix.

TASEP is a model consisting of a many-particle hopping system where particles are located on a discrete lattice that evolves in continuous time. Particles can hop to the next lattice site, in only one direction (say to the right-hand side), on a one-dimensional lattice at a random time, with rate one, provided that the target site is empty. We here consider the problem with open boundary conditions where both sides of the lattice are coupled with particle reservoirs. If the first site of the lattice is empty then a particle can hop from the reservoir into the system with a transition rate *α* and the particles leave the system from the last site of the lattice with a transition rate *β*. TASEP has been used to describe directed transport in 1D, such as arises, for instance, in unidirectionally moving vehicular traffic along roads^7^,^8^.

There are several reasons to choose TASEP for the present study: The equal-time correlation functions for this stationary non-equilibrium system are known exactly^9^,^10^ and the phase diagram (see Fig. 1) of the exact and the mean field solutions coincide.

The phase diagram is shown in Fig. 1: in phase H, there is a high density of occupied sites which fluctuates little in time, in phase L there is a corresponding low-density phase, in which the density is also approximately time-independent, whereas in phase MC the density is equal to 1/2, independent of *α* and *β*. Finally, in the transition line between H and L, a phase exists in which the density oscillates between a high value corresponding to a nearby point of phase H, and a low value corresponding to a nearby point of phase L.

We are interested in the density-density two point correlation *C*_*i,j*_ on the lattice. This is defined as the probability to find a particle in a lattice site *j*, given there is a particle at lattice site *i*. The analytical expression for this correlation function *C(r*) (average correlation between any two points at distance *r*) is given in ref. 10. This correlation function is everywhere found to be negligible, except on the line *α* = *β* < 0.5, where both the high density and low density phase coexist: there the two point correlation function decays more rapidly than exponential, yet ranges over distances of the order of the system size. This anomalous behaviour is illustrated in Fig. 2.

The results we report are then as follows: we observe that the eigenvalues of the correlation matrix *C*_*i,j*_ display a behaviour very different from that expected for a random series. The latter is the so-called Marčenko–Pastur (MP) law for the density of eigenvalues, which predicts that the eigenvalues are continuously distributed within a finite interval and with a given density. The kind of deviations we observe are of two kinds: first, the existence of relatively few eigenvalues that are much larger than what is expected from MP, and which are distributed according to a power-law. Second, we observe a far larger number of eigenvalues which are significantly lower than what would be expected from MP, and their number grows as 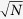, where *N* is the length of the model. We proceed to describe these phenomena in greater detail.

## Results

Let us start by comparing the eigenvalue density of the correlation matrix of the TASEP model we study, with the null-hypothesis, that is, the eigenvalue spectrum of the correlation matrix of a completely uncorrelated signal. These correlations remain different from zero if they are taken on the scale of the fluctuations, that is, on the scale of the square root of the duration of the signal. The eigenvalue distribution may be calculated exactly in this case, and the eigenvalue density was determined analytically by Marčenko and Pastur^11^. This eigenvalue density has the remarkable feature that it vanishes outside a finite interval. We may thus meaningfully speak of deviations from the Marčenko–Pastur (MP) result whenever eigenvalues appear significantly outside this interval.

In the following we analyse the system’s behaviour in the various regions of the phase diagram. We thus compare our eigenvalue spectra with the MP distribution in order to see to what extent our eigenvalues differ from an uncorrelated signal. This is very much in the line of refs 2,3. Our key result can now be stated as follows: we find agreement in the high and low density regions, that is, in the interior of regions H and L, so that these are indeed well described by a random process. On the other hand, in all other parts of the phase diagram, characteristic differences are observed.

**Figure 1:**
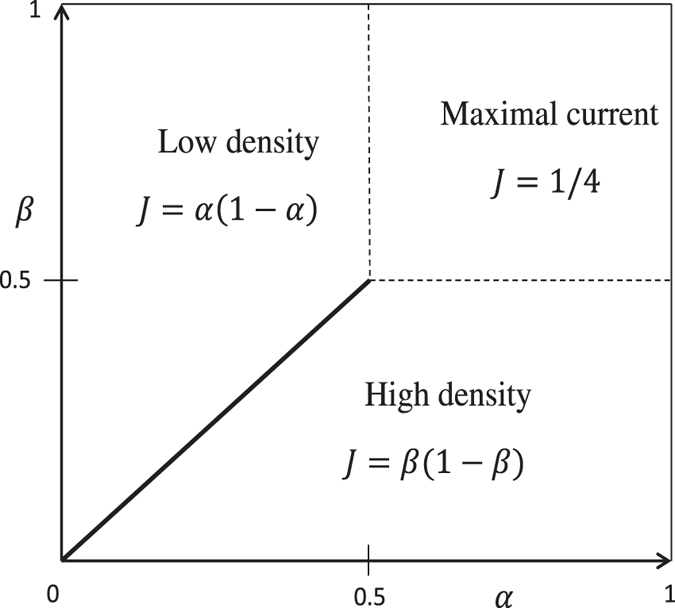
Phase diagram of TASEP with open boundary conditions at the thermodynamic limit, consisting of the high-density phase (phase H), the low-density phase (phase L) and the maximum-current phase (phase MC). Inside each phase *J* denotes the current in the stationary state, which must be independent of position.

There are three pure phases (see Fig. 1) and three coexistence lines. However, since the system has a symmetry between holes and particles, the High density phase (phase H) is equivalent to the Low density phase (phase L), and the coexistence line of high density–maximal current (H/MC) line is equivalent to low density–maximal current (L/MC) line. Looking at phase H, we numerically confirm full agreement (picture not shown) between the eigenvalues of the correlation matrix and MP. Further, the H/MC coexistence curve behaves (apart from some minor issues, to which we shall return) similarly to the maximum current phase; we are therefore left with two regions to consider: (1) the high density–low density coexistence line (H/L coexistence line), (2) the maximum current phase (MC). We present the results for each case separately.

### The H/L coexistence line

We shall analyse the eigenvalues using the so called Zipf plot, also known as “scree diagram” or ranking-of-eigenvalues plot, in which the eigenvalues in decreasing order *λ*_*n*_ are plotted against their rank *n*, typically on a doubly logarithmic plot. Such a plot makes an initial power-law very prominent.

**Figure 3:**
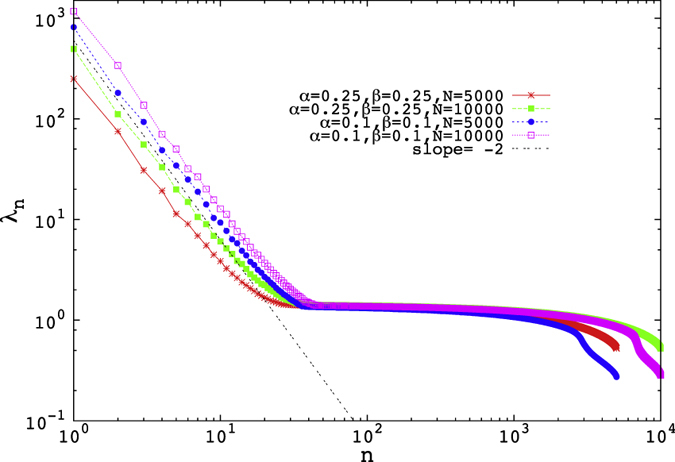
The Zipf plot for the ranked eigenvalues for different values of *α* and *β* on the low density-high density coexistence line (*α* = *β* line) .

For *α* = *β* < 0.5 we find such a power-law (Fig. 3) in the Zipf plot (see below) and thus an example, where we find in the eigenvalues a behaviour that is not found in the two-point function in space. The power we find (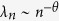 with *θ* ≈ 2) obtained is same for any value of *α* and *β*, as long as *α* = *β* < 0.5 [Fig. 3].

**Figure 4:**
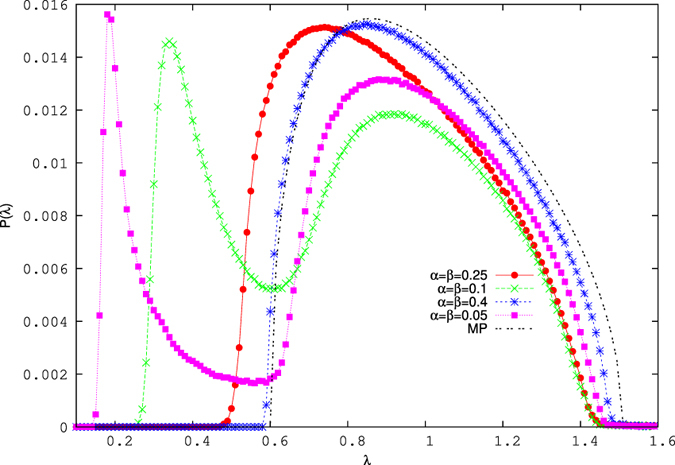
The plot of bulk for the distribution of eigenvalues for different values of *α* and *β* on the low density-high density coexistence line (*α* = *β* < 0.5 line). The MP distribution is shown by the black double dashed line.

There are other differences between the observed distribution on the H/L separation line and the MP distribution: first, the range over which the power-law is observed, varies with the parameter value. It is higher for the lower values of *α* = *β*. As a result, the density of high-lying eigenvalues differ for different values of *α* and *β* on this line. Second, we observe a shift of the bulk to lower eigenvalues in compared with the MP distribution [Fig. 4]. This shift actually compensates the contribution from the higher eigenvalues, since the sum of the eigenvalues remains constant and equal to the dimension of the matrix. However for lower values of *α*, the density profile for the eigenvalues are deformed and the deformation becomes more prominent as the value of *α* = *β* decreases [Fig. 4]. Finally, when *α* ≲ 0.25, a side peak in the eigenvalue density appears at small eigenvalues, in sharp contrast to the case in which 0.25 ≲ *α* = *β*, in which only one peak (main peak) appears.

This phenomenon may perhaps be explained as follows: the density of particles inside the lattice, in the low density region is lower for the lower values of *α*. Similarly it is higher for the lower values of *β*, in the high density region. Hence for *α* = *β* < 0.5, that is on the H/L coexistence line, for the lower values of *α* and *β* there will be larger strings of particles in the lattice followed by a string of empty lattice sites of similar length. As a result correlation length inside the lattice increases as *α* decreases (for *α* = *β* < 0.5). This correlation length is not enough to show a power law decay in case of two point correlation function, but it may well be related to the presence of a larger number eigenvalues above the MP threshold. These then display the power law behaviour observed in the Zipf plot. Note that the number of these large eigenvalues is quite insensitive to the system size, growing from approximately 20 for *N* = 500 to about 40 for *N* = 10000, meaning that we cannot, with present computational power, reliably determine the *N*-dependence of this number.

Apart from the eigenvalue density, there exists another set of quantities referring to the fine scale of the eigenvalue spectrum, namely the correlations between two eigenvalues. It has been shown in a broad variety of cases, see for example^12^, that for random matrix ensembles of the type considered here, these properties are *universal*, that is, they are the same for an very broad class of random matrix models. Since the side peak in the distribution is quite unexpected, we now look at these features, to see whether it is different from the main peak in some essential manner. We thus consider the side peak in the eigenvalue density that appears for *α* = *β* ≲ 0.25. In particular, it is natural to ask whether, inside the two peaks of eigenvalue density which arise for lower values of *α* and *β*, the correlations of the unfolded eigenvalues (say *ξ*)^12^, are identical. We test two independent statistical properties of unfolded eigenvalues *ξ*: the distribution of nearest-neighbour spacing *s* = *ξ*_*i*+1_ − *ξ*_*i*_ and the statistics of number variance 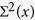.

The distributions of nearest-neighbour spacing of unfolded eigenvalues, which are obtained from the first and the side peak of the eigenvalue density [Fig. 4] appear to be universal. That means the behaviour of the nearest-neighbour (nn) spacing distributions are not distinguishable from that of the Wishart ensemble for both the peaks.

*Number variance*, the variance of the number of unfolded eigenvalues in the intervals of length *x*, is defined as 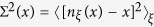, where 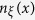 is the number of unfolded eigenvalues in the interval [*ξ* − *x*/2, *ξ* + *x*/2]. The average is made along *ξ*. If the eigenvalues are uncorrelated, 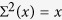; whereas if all unfolded eigenvalues are equidistant, 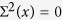.

The eigenvalues manifestly belonging to the peak were first unfolded (in other words, reduced to constant density by an appropriate smoothing transformation) then the central 60% of the eigenvalues were kept, leading to 400 eigenvalues for the side peak at *α* = *β* = 0.1 and 1600 for the main peak. In the case of *α* = *β* = 0.05 we were left with 150 eigenvalues for the side peak and again 1600 for the main peak. This means that finite size effects are significantly stronger in the latter case than in the former. This might then explain the discrepancy between 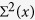 for the two peaks, but there could be a deeper reason for it. It is in any case a remarkable fact that the deviation in the case *α* = *β* = 0.05 is quite similar to the deviation observed in quantum spectra of classical chaotic systems^13^, which is known to be caused by the existence of a shortest periodic orbit^14^.

**Figure 5:**
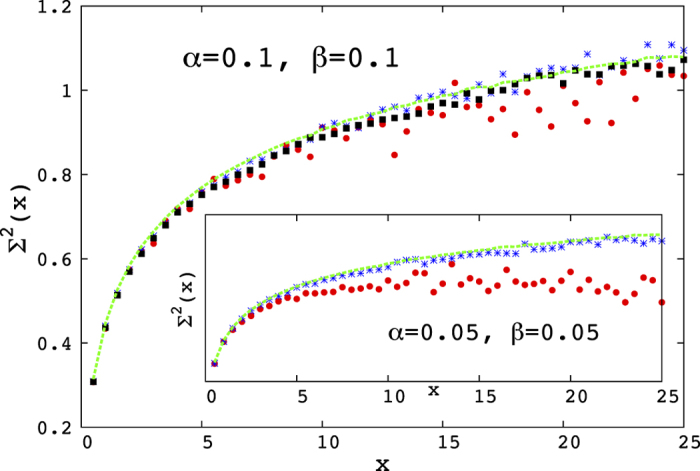
The number variance ∑^2^(*x*), is plotted against the interval length *x*, calculated separately from the side peak (red points) and the main peak (blue stars) of their eigenvalue density for points *α* = *β* = 0.1. For *α* = *β* = 0.4 (black squares) ∑^2^(*x*) is calculated from the single bulk of its eigenvalue density. The latter fits well to the continuous line, which is a Wishart ensemble of 2000 configurations, plotted for comparison. The inset shows ∑^2^(*x*) for both peaks at *α* = *β* = 0.05 as well as the Wishart ensemble for comparison.

We found the unfolded eigenvalues obtained from the side peak of the distribution does not follow the universal behavior for the number variance statistics, while for the main peak it appears to be universal [Fig. 5].

### The maximum current phase

**Figure 6:**
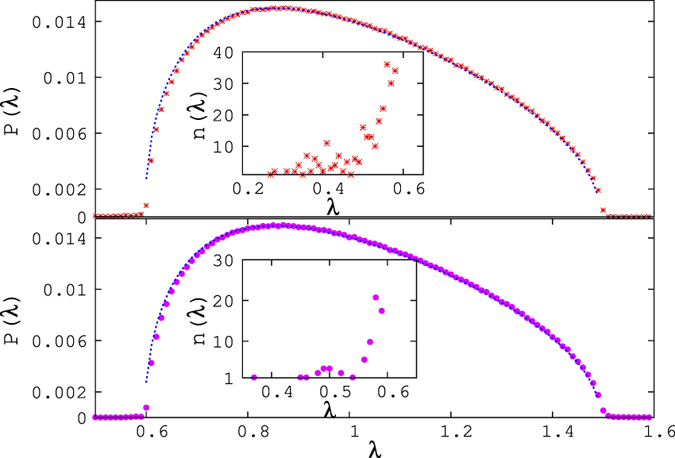
The probability distribution of eigenvalues at the maximal current (upper panel) regime and for the triple point (lower panel). The MP distribution is shown by the blue dotted line. Inset shows the number distribution of below threshold eigenvalues. In MC region, the number of eigenvalues below the lower threshold of MP is significantly more than that of the Triple point. Both of these results were obtained with 60 samples of size 5000.

**Figure 7:**
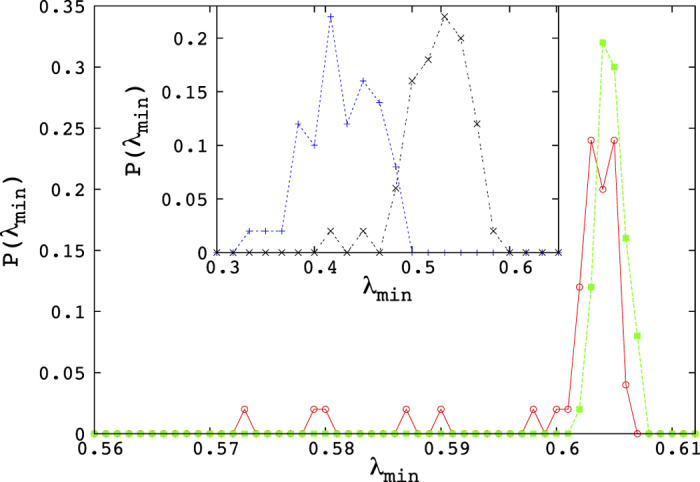
Histogram of the lowest eigenvalue of the correlation matrices for different parts of the phase diagram. We took 100 samples for each curve. The green (squares) corresponds to the H phase, the red (circles) to the triple point, whereas, in the inset, the black (cross marks) corresponds to the H/MC coexistence line and the blue (plus marks) to the MC phase. Note that the green curve is very strongly peaked, corresponding to the well-known fact that the lower end of the MP eigenvalue density is very sharp. This figure shows the deviations from this behaviour in the other regions of the phase diagram. As we see, the triple point has a few, but probably a significant number of, eigenvalues lower than this threshold. On the other hand, both the MC phase and the H/MC coexistence line have a large number of such eigenvalues lying far below the threshold.

In the maximal or constant current region, we also find significant deviations from MP in the probability distribution of eigenvalues [see inset of Fig. 6 as well as Fig. 7]. This consists in the appearance of eigenvalues below the lower threshold of MP, and is quite pronounced.

**Figure 8:**
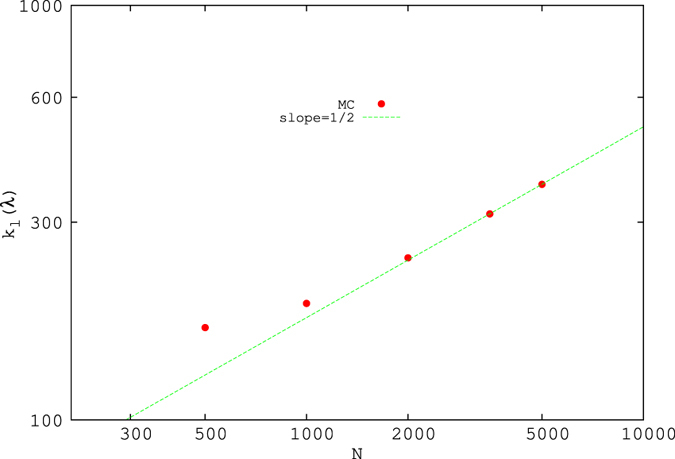
Number of eigenvalues lying below the MP distribution in the maximal current phase, as a function of system size. A line corresponding to 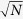 is also drawn.

One or two high eigenvalues (outside the limits of MP) are also observed. The plots for the eigenvalue density for the maximal current and for the triple point are shown in Fig. 6. Transition from the MC phase to the triple point is continuous, as the number of lower eigenvalues decreases slowly as the triple point is approached. We have also observed that the total number of lower eigenvalues (*k*_*l*_(*λ*)), below the lower threshold of MP distribution increases as a function of system size. 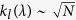 for large *N*, where *N* is the size of the system [see Fig. 8].

The deviation of the probability distribution of eigenvalues of the correlation matrix is also present on the transition lines of H/MC and L/MC. But there the number of below-threshold eigenvalues distribution is smaller than in the constant current phase.

In this part of the phase diagram, namely the MC phase proper, the H/MC coexistence line, as well as the triple point, the probability densities of the eigenvalues have a deviation from the Marčenko–Pastur but also the distribution of the lowest eigenvalues, over the configuration space is significantly different from that of the low density or high density regions [Fig. 7].

## Discussion

Summarising, we have found that the eigenvalues of the TASEP correlation matrices display remarkable properties that differ from those characteristic of random signals, even though the TASEP correlations are small, and surely not power-law. We observe in particular a relatively small number of large eigenvalues, distributed according to a power-law, on the H/L coexistence line. Additionally, we observe deviations from the MP eigenvalue density, which are particularly pronounced when *α* = *β* ≲ 0.25, where two peaks are observed. In the MC phase, on the other hand, we observe good agreement between MP and the observed eigenvalue density, apart from a set of eigenvalues lying below the lower limit of MP, the number of which is found to grow as the square root of the system size.

We have also checked whether the observations can be partly accounted for by edge effects: we did not notice any significant such effect for any value of *α* and *β* for the entire phase diagram. This is also true for the higher eigenvalues when *α* = *β* < 0.5. If there is any indication of such behaviour at all in the spectrum of eigenvalues, it is not detectable with the present computational accuracy.

On the H/L phase coexistence line, we have taken different parts of the lattice and repeated the correlation matrix analysis. We indeed observed the power law in the Zipf plot (with the same value of *θ*) for all the parts of the lattice which will be discussed in detail at ref. 15. On this coexistence line the motion of the domain wall is non-localised over the lattice^16^. Whether or not the motion of the domain wall is responsible for the observed power law will be studied in future^15^. We will also attempt to connect the formula of two point function given in ref. 10 to the exact solutions derived recently for arbitrary correlations at least in an approximate fashion.

In conclusion, we have shown that the analysis of the density of eigenvalues of the correlation matrix of a signal is sensitive to non-trivial correlations, which cannot otherwise be reliably characterised by direct numerical observation. Such is the case of TASEP, in which two-body correlations are weak, though they extend over the whole system at the phase coexistence line. Comparison of the correlation matrix spectrum with those generated by a random signal provide clear evidence that the signal produced by TASEP has significant correlation in some parts of the phase diagram. In particular at the *α* = *β* < 1/2 line long-range weak correlations in space induce a power law in the spectrum.

## Methods

To construct the correlation matrices, we have generated the times series by Monte-Carlo simulation. The random update rule was been used to generate the time series. The lattice size is *N*, with 5 × 10^2^≤ *N* ≤ 10^4^. For each parameter value we have considered the length of the time series as *T* = 20 × *N*. Obviously the correlation matrix *C* is an *N* × *N* dimensional matrix. The results are averaged over an ensemble of 100 configurations.

## Additional Information

**How to cite this article**: Biswas, S. *et al*. Rich structure in the correlation matrix spectra in non-equilibrium steady states. *Sci. Rep.* **7**, 40506; doi: 10.1038/srep40506 (2017).

**Publisher's note:** Springer Nature remains neutral with regard to jurisdictional claims in published maps and institutional affiliations.

## Figures and Tables

**Figure 2:**
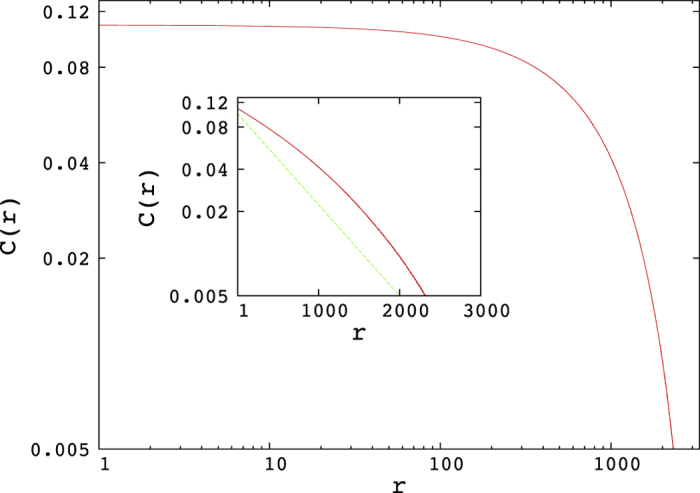
The two-point correlation function for the TASEP on the H/L coexistence line, specifically for *α* = *β* = 0.25 and for *N* = 5 · 10^3^. The main figure shows a log-log plot, which clearly shows that there are no power-law correlations, whereas the inset shows a log-linear plot, indicating that the decay is faster than exponential. The scale of the *x* axis indicates that the correlations range over the whole system size.
